# Genomic and regulatory characteristics of significant transcription factors in colorectal cancer metastasis

**DOI:** 10.1038/s41598-018-36168-8

**Published:** 2018-12-13

**Authors:** Bin Zhou, Rui Guo

**Affiliations:** 1 School of Life Science, Tsinghua University, Beijing, 100084 China; 2 Department of Biochemistry and Molecular biology, Shanxi Medical University, Taiyuan, China

## Abstract

The dysregulation of transcription factors has an important impact on the oncogenesis and tumor progression. Nonetheless, its functions in colorectal cancer metastasis are still unclear. In this study, four transcription factors (HNF4A, HSF1, MECP2 and RAD21) were demonstrated to be associated with the metastasis of colorectal cancer in both RNA and protein levels. To comprehensively explore the intrinsic mechanisms, we profiled the molecular landscape of these metastasis-related transcription factors from multiple perspectives. In particular, as the crucial factors affecting genome stability, both copy number variation and DNA methylation exerted their strengths on the expression of these transcription factors (except MECP2). Additionally, based on a series of bioinformatics analyses, putative long non-coding RNAs were identified as functional regulators. Besides that, rely on the ATAC-Seq and ChIP-Seq profiles, we detected the target genes regulated by each transcription factor in the active chromatin zones. Finally, we inferred the associations between the target genes by Bayesian networks and identified LMO7 and ARL8A as potential clinical biomarkers. Taken together, our research systematically characterized the regulatory cascades of HNF4A, HSF1, MECP2 and RAD21 in colorectal cancer metastasis.

## Introduction

Colorectal cancer is a kind of malignant human tumor with high mortality around the world. The incidence of colorectal cancer shows a gender difference and is prone to the elderly. The pathogenesis of colorectal cancer is complex, and some factors may increase the risk, such as inflammatory bowel disease, race, smoking history, dietary composition and so on. Until now, tumor metastasis remains the dominant lethal factor of colorectal cancer that cannot be effectively controlled. In view of the highly heterogeneity of colorectal cancer metastasis, it is urgent to identify molecular targets for clinical treatment. Due to that, the role of transcription factors in colorectal tumor metastasis has been increasingly emphasized^1,2^, and their dysregulations can result in disorders of intracellular regulatory networks, which may further bring about the chaos of target genes expression.

As the core transcription factors in our study, HNF4A, HSF1, MECP2 and RAD21 were all validated to be relevant to the tumorigenesis and development of colorectal cancer. In detail, the function of HNF4A (hepatocyte nuclear factor 4 alpha) in colorectal cancer was controversial. By animal experiments, Darsigny *et al*. found that HNF4A can facilitate gut neoplasia and protect cancer cells against reactive oxygen species^3^. Interestingly, Vuong *et al*. reported that different isoforms of HNF4A exerted distinct functions in colon cancer for their different manners of interaction with Wnt/β-catenin/TCF4 and AP-1 pathways^4^. HSF1 is a heat shock transcription factor, which is sensitive to environmental temperature stress. In colorectal tumors, DAPK can phosphorylate HSF1, meanwhile, HSF1 can increase the expression of DAPK. Such positive feedback can promote the TNF-induced apoptosis^5^. Furthermore, Mendillo *et al*. found HSF1 was highly activated in multiple types of human malignant tumors (including colorectal cancer) and markedly affected the transcriptome networks in cancer cells^6^. As a member of methyl-CpG binding protein family, MECP2 broadly participated in regulating the expression of methylated genes. *In vitro* experiments demonstrated that knockdown of MECP2 would lead to the growth inhibition of colorectal cancer cells, which implied the facilitating effect of MECP2 on carcinogenesis^7^. For RAD21, as a component of cohesion complex, it widely involved in multiple cellular process, including DNA repair, cell cycle, apoptosis and so on. In colorectal tumor, Xu *et al*. found RAD21 was upregulated due to the aberrant activation of Wnt signaling pathway^8^. Although the roles of these transcription factors in colorectal cancer have been noted, their influences on tumor metastasis are still vague.

In our study, the transcription factors HNF4A, HSF1, MECP2 and RAD21 showed differential expression on both RNA and protein levels in colorectal cancer metastatic patients compare to the non-metastatic group. To find out the sources of such disorder, we examined the variations of each TF in different levels. In addition to MECP2, both copy number variation and DNA methylation had influences on the RNA expression of HNF4A, HSF1 and RAD21 to some extent. Not only that, we also identified putative lncRNA regulators of HNF4A, HSF1 and MECP2, which may affect their functions. On the other hand, ChIP-Seq profiles of each transcription factor, combined with the ATAC-Seq profiles, were used to detect the downstream target genes with high reliability. Furthermore, we constructed the Bayesian networks to infer the associations between downstream target genes, and screened potential biomarkers by machine learning tools and statistical methods. In conclusion, our analyses profiled the regulatory axis around four metastasis-related transcription factors, which can provide novel insights into the pathogenesis of colorectal cancer metastasis.

## Results

### The overview of the analyses pipeline

**Figure 1:**
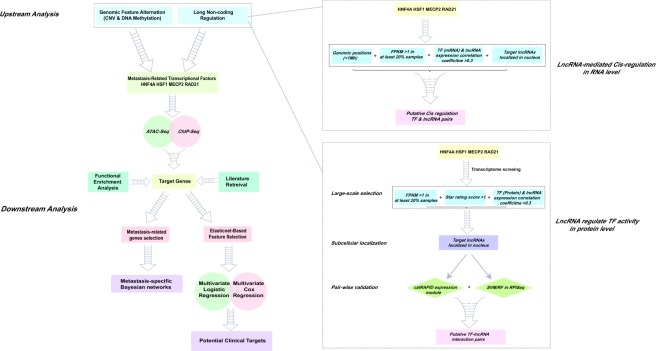
The computational analyses workflow for exploring significant transcription factors in colorectal cancer metastasis.

The overall scheme of our study was illustrated in Fig. 1. Firstly, colorectal cancer metastasis-related transcription factors (TF) were identified through the Wilcoxon rank sum test on both transcriptomic and proteomic profiles of patients in TCGA database.

Next, to investigate the possible causes of such dysregulation, we analyzed the variations of upstream genomic features, and identified potential long non-coding RNA regulators of these TFs in RNA and protein levels. Thirdly, integrative analyses were implemented to explore the biological roles of downstream target genes in colorectal cancer metastasis.

### Identification of significant transcription factors relevant to colorectal cancer metastasis

As crucial regulatory proteins, transcription factors (TFs) play a crucial role in colorectal cancer metastasis^9,10^, and thus the alteration of their expression patterns between metastatic and non-metastatic patients can reflect the changes of their corresponding biological functions. Here, we mainly used the proteomic and transcriptomic datasets (see Material and Methods) to identify important TFs that are associated with colorectal cancer metastasis. In particular, we first selected 140 TFs with the LC-MS/MS-based proteomic profiles of 90 colorectal cancer patients (11 with metastasis) from the Clinical Proteomics Tumor Analysis Consortium (CPTAC)^11^. Next, among these 140 selected TFs, we identified 18 TFs that displayed significant different protein expression values between metastatic and non-metastatic colorectal cancer patients (P value < 0.05, Wilcoxon rank sum test; Supplementary Table S1).

Next, we used the RNA-Seq profiles of 598 colorectal cancer patients in TCGA database, which contained 88 patients with cancer metastasis (see Material and Methods), to further filter the above obtained 18 TFs. In particular, we found that eight among these 18 TFs exhibited significantly different transcriptomic expression values between metastatic and non-metastatic groups (P values < 0.05, Wilcoxon rank sum test; Supplementary Table S1). Through the two steps, we identified the transcription factors with differential expression in both protein and RNA levels, which implied their essential actions in colorectal cancer metastasis.

**Figure 2:**
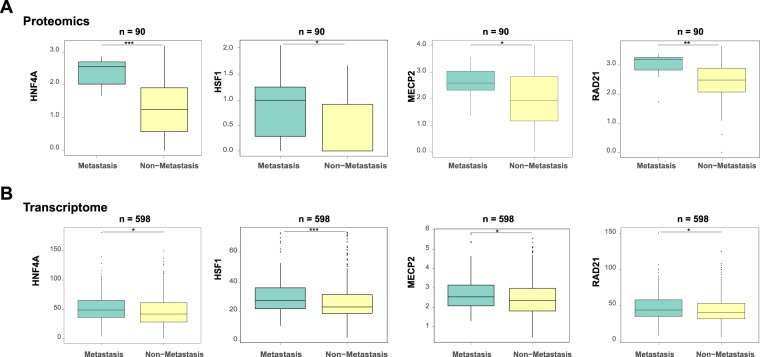
Differential expression distributions of the identified transcription factors (i.e., HNF4A, HSF1, MECP2 and RAD21) between metastatic and non-metastatic colorectal cancer patients, in terms of the protein abundance from proteomics profiles (**A**) and the RNA expression values from transcriptomic profiles (**B**). *P value < 0.05, **P value < 0.01, ***P value < 0.001, two-sided Wilcoxon rank sum test.

To enable the downstream analysis of regulatory targets of TFs, we only focused on the four among the above eight TFs, including HNF4A, RAD21, MECP2 and HSF1 (Fig. 2A,B), which had the available and high quality ChIP-Seq datasets derived from the colorectal cancer cells in the Cistrome Data Browser^12^.

### Genomic feature variations of the identified transcription factors

In tumor cells, genomic state alterations were known as one of the important factors influencing related gene expression patterns, such as copy number variation and DNA methylation level^13,14^. In view of this, the functions of genomic features in the dysregulation of these previously identified TFs (i.e., HNF4A, HSF1, MECP2 and RAD21) should not be neglected.

**Figure 3:**
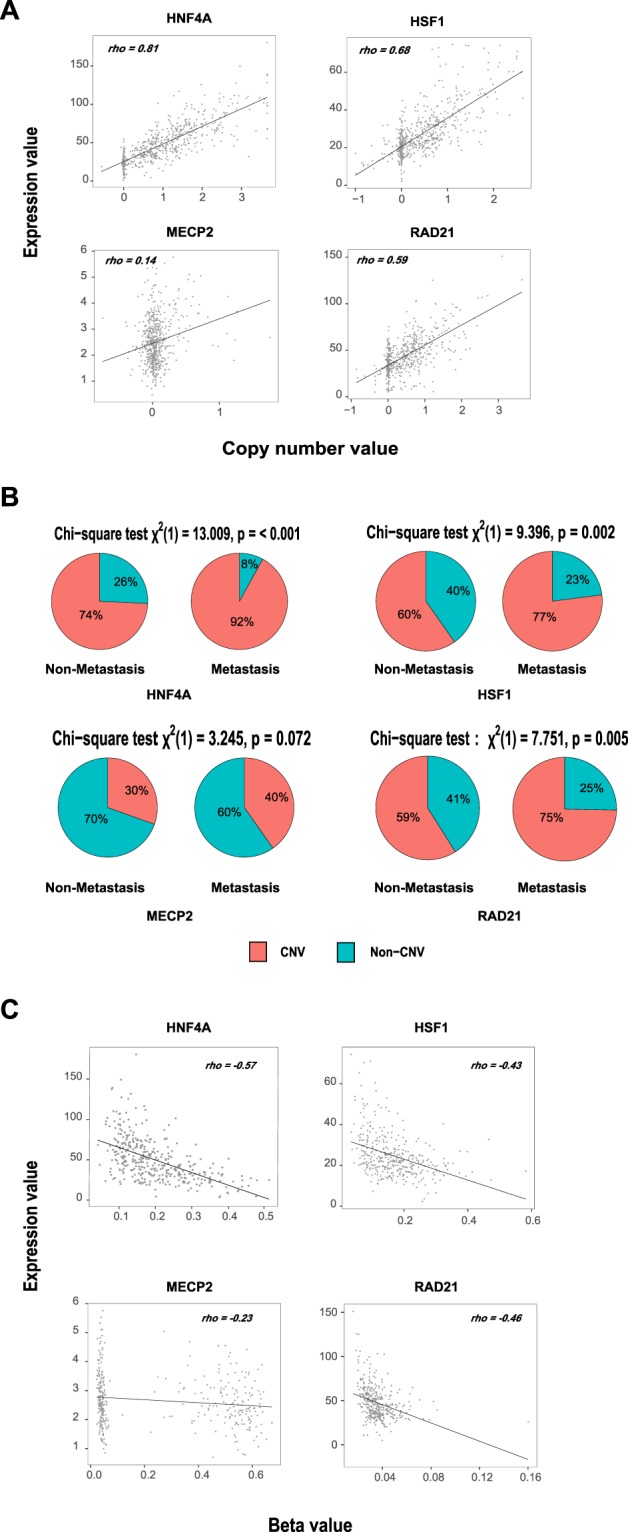
Genomic feature variations for understanding the gene expression dysregulations of HNF4A, HSF1, MECP2 and RAD21. (**A**) The Spearman correlations between the copy number values and FPKMs of HNF4A, HSF1, MECP2 and RAD21. (**B**) Chi-square test on the copy number variation distributions in non-metastatic and metastatic patients for each transcription factor. (**C**) The Spearman correlations between DNA methylation beta values and FPKMs of HNF4A, HSF1, MECP2 and RAD21.

Through GISTIC 2.0 algorithm^15^, we identified the recurrent copy number variations of each colorectal cancer patient (see Material and Methods). We then extracted the copy number values of HNF4A, HSF1, MECP2 and RAD21 in all samples (Supplementary Table S2), and examined the influence of CNVs on the gene expression of these four TFs. We found that the CNVs of HNF4A, HSF1 and RAD21 were highly correlated with their gene expression (Spearman correlations above 0.59), while MECP2 displayed a relatively weak trend (Spearman correlation 0.14) (Fig. 3A). In addition, from the discretized CNVs of the four TFs in all patients (Supplementary Table S2), we compared the ratio of patients suffered CNVs between non-metastatic and metastatic groups for each transcription factor. Aligned with the previous results on the correlations between CNVs and gene expressions, all four TFs except MECP2 displayed significant different fractions of patients with CNVs between non-metastatic and metastatic groups (P value < 0.05, Chi-square test; Fig. 3B).

On the other hand, we collected the beta values of the selected DNA methylation probes for each TF (see Material and Methods; Supplementary Table S2). After calculating the Spearman correlation coefficients between the beta values and corresponding gene expression of each TF, we observed that DNA methylation level of HNF4A, HSF1 and RAD21 had more influence on their RNA expression than MECP2 (Fig. 3C). However, the DNA methylation states do not demonstrate significant differential distributions between non-metastatic and metastatic groups like copy number variations through Wilcoxon rank sum test.

To sum up, both copy number variation and DNA methylation have relatively strong effects on the expression of HNF4A, HSF1 and RAD21 at RNA level. It is worth noting that, from our results, copy number variation may contribute to the dysregulations of these transcription factors (except MECP2) in colorectal cancer metastasis.

### Detection of potential cis-regulatory long non-coding RNAs

Long non-coding RNAs (lncRNAs) are RNA transcripts longer than 200 bp but cannot be translated into proteins. With the in-depth studies, increasing experimental evidences revealed the role of lncRNAs in regulating genes expression in cells. Due to the diversity of structures and subcellular localizations, the regulatory mechanisms varied widely among different types of lncRNAs, which spanned multiple molecular levels. In particular, lncRNAs can influence the chromatin structures through recruiting chromatin remodeling factors^16^, affecting DNA methylation^17^ and histone modification state^18^, and so on. In addition, lncRNAs also involved in the post-transcription regulation of mRNAs, including alternative splicing^19^, translation^20^, and protein modification^21^. On the other hand, the genomic localizations of lncRNAs were also a critical factor affecting their regulations, and some lncRNAs tended to regulate the expression of the neighboring genes^22^, which was defined as the cis-regulation.

In the previous section, we analyzed the effect of genomic features on the expression of HNF4A, HSF1, MEPC2 and RAD21. Here, we aimed to detect the lncRNAs that acted as the cis-regulators for these TFs, and the analysis procedure was depicted in Fig. 1. The genomic position of each transcription factor was set as the center, and we searched the lncRNA candidates within the range of 1Mb around it. In the results, for filtering out the low expressed lncRNAs, the ones with FPKM >1 in at least 20% samples were retained. After calculating the Spearman correlation coefficients of the FPKMs between each TF and their lncRNA partners, we set 0.3 as the threshold and obtained ten TF-lncRNA pairs with coefficients >0.3 (Supplementary Table S3). Moreover, considering the cis-regulation mainly occurred in the nucleus, we also queried the subcellular localizations of the ten lncRNAs in lncATLAS^23^, and found seven of them had the distribution signals in nucleus (Supplementary Table S3).

To sum up, according to the mechanism of lncRNAs cis-regulation, we integrated multiple information, including genomic positions, genes expression and subcellular localizations, to detect the potential lncRNA regulators that may influence the expression of the transcription factors in RNA level.

### Putative long non-coding RNAs that regulate the activities of transcription factors

Although the role of lncRNA in regulating gene expression is indubitable, it needs the cooperation of specific proteins in many cases. Therefore, identifying the functional lncRNA-protein interactions has become an effective way to reveal the intrinsic molecular mechanism of lncRNA regulations. Notably, lncRNAs can interact with transcription factors and affect their functions^24^, which provided a novel view to understand the cellular transcription regulations. Here, we attempted to explore the putative lncRNA regulators of HNF4A, RAD21, MECP2 and HSF1 in protein level.

Despite relevant high-throughput sequencing technologies (like CLIP-Seq *etc*.) have been used to detect the RNA partners for specific proteins in the whole genome, such studies mainly focused on RNA-binding proteins, not transcription factors, that is, there were rare deep-sequencing datasets on detecting large-scale TF-lncRNA interactions. In view of this, it is a fast and effective way to predict the TF-lncRNA interactions by accepted bioinformatics algorithms. As the scheme described in Fig. 1, we performed multiple procedures to detect the putative lncRNA partners for each transcription factor.

Initially, based on the protein sequence information of HNF4A, HSF1, MEPC2 and RAD21, we gained the TF-lncRNA interactions pooling in whole transcriptome level through *catRAPID omics*^25^ (see Material and Methods). Next, we further determined the high credible lncRNA candidates according to the following criteria: (1) the FPKM of each lncRNA candidate should be greater than 1 in at least 20% samples. (2) For each TF-lncRNA pair, its star rating score (the comprehensive index for evaluating interaction status in *catRAPID omics*) should be greater than 1 (moderate level). (3) The Spearman correlation coefficient between protein expression value of each TF (from the proteomics dataset) and the FPKM of its lncRNA partner should be greater than 0.3.

Through the above multi-step screening, 14 putative lncRNA partners were identified for HNF4A, 3 ones for MECP2 and 2 ones for HSF1 (Supplementary Table S4). RAD21 was eliminated for all of its predictive lncRNA partners had very low star rating scores (less than 1).

Considering transcription factors generally exerted functions in cell nucleus, it is necessary to further identify the members of the putative lncRNAs which had the same subcellular localization. Through searching in the lncATLAS database^23^, we retained the lncRNA candidates that can be detected preferential nuclear position signals: SNORD12C, RP11-473M20.9, SNORA5A, LINC01138, SNHG11, SNHG15, TPT1-AS1, LINC00265, RP11-44N21.1, CTD-2377D24.6, LINC01006, SNHG17 and CTB-31O20.2. And then, each interaction pair was analyzed by the *catRAPID express*^26^ module respectively, which can provide more details based on the sequence features of TFs and their partner lncRNAs.

**Figure 4:**
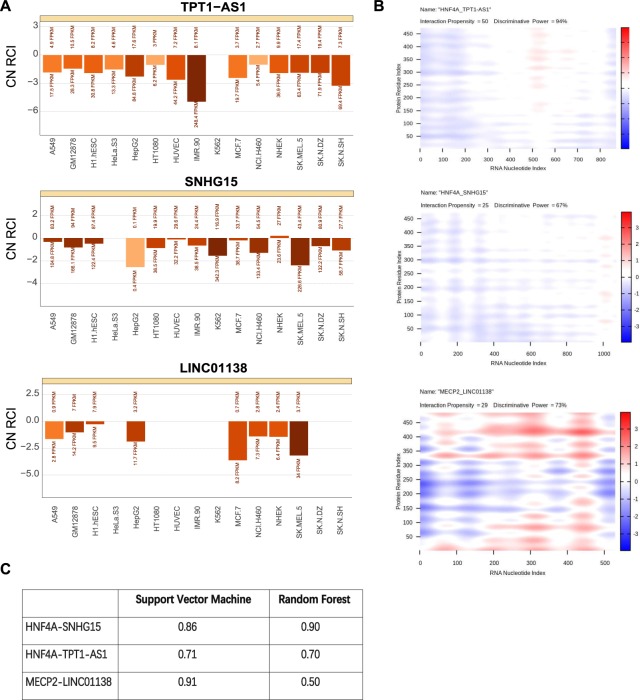
Three potential regulatory long non-coding RNAs identified through the bioinformatics analysis pipeline. (**A**) The subcellular localizations of the identified lncRNAs TPT1-AS1, SNHG15 and LINC01138 (retrieved from lncATLAS^23^). The x axis represents different cell lines and y axis represents the relative concentration index (RCI). (**B**) The heatmap shows prediction results of protein-lncRNA interactions in *catRAPID express* module. The colors denote interaction strength of each amino acid and nucleotide pair. (**C**) The prediction results generated by RPISeq. The values in the table indicate the interaction probabilities of HNF4A-SNHG15, HNF4A-TPT1-AS1, MECP2-LINC01138.

After that, three pairs were identified with obvious evidence: HNF4A-TPT1-AS1, HNF4A-SNHG15 and MECP2-LINC01138 (Fig. 4A,B). All of the three pairs had high interaction propensity, and their discriminative power were all greater than 50%, which indicated the high probability of interactions. Additionally, we found MECP2-LINC01138 displayed obvious regional interaction characteristics, compare to the overall interaction patterns of HNF4A-SNHG15 and HNF4A-TPT1-AS1 pairs (Fig. 4B).

For further increasing the reliability of the results, we verified these pairs on RPISeq algorithm^27^, which was based on support vector machine and random forest classification. And it defined that predictions with interaction probabilities more than 50% to be considered meaningful. All of the three pairs had good performance on this algorithm (Fig. 4C).

In conclusion, we identified three putative protein-lncRNA regulatory pairs by multiple strict standards. The whole process involved both the sequence and expression information. Different algorithms cross validated the results from different perspectives, thus further enhancing their credibility.

### Detection of target genes based on ATAC-Seq and ChIP-Seq profiles

To further analyze the underlying influence of transcription factors on colorectal cancer metastatic gene expression, we systematically parsed their downstream target genes in whole genomic level. The ChIP-Seq profiles of HNF4A, HSF1, MECP2 and RAD21 were all collected from Cistrome Data Browser (see Material and Methods). Comprehensive considering the datasets quality and the source of cancer cell lines, we chose the ChIP-Seq datasets of MECP2 and RAD21 derived from HCT116 cell line, HSF1 derived from HT29 cell line, and HNF4A derived from LoVo cell line (Supplementary Fig. 1A–D). These cell lines stem from malignant colorectal cancer tissues, which can better reflect the biological characteristics of cancer cells.

In addition, another question worth pondering is the open state of chromatins for transcription factors binding. The ATAC-Seq can detect the open DNA regions in the whole genome level, which provided the information on active transcriptional states in cells. Here, we adopted the ATAC-Seq profiles derived from HCT-116 cell line and identified associated genes in active chromatin regions (see Material and Methods). Except distal intergenic, half of peaks were clustered in gene promoter regions, especially, <1 kb distance from transcriptional start site (TSS) (Supplementary Fig. 1E).

**Figure 5:**
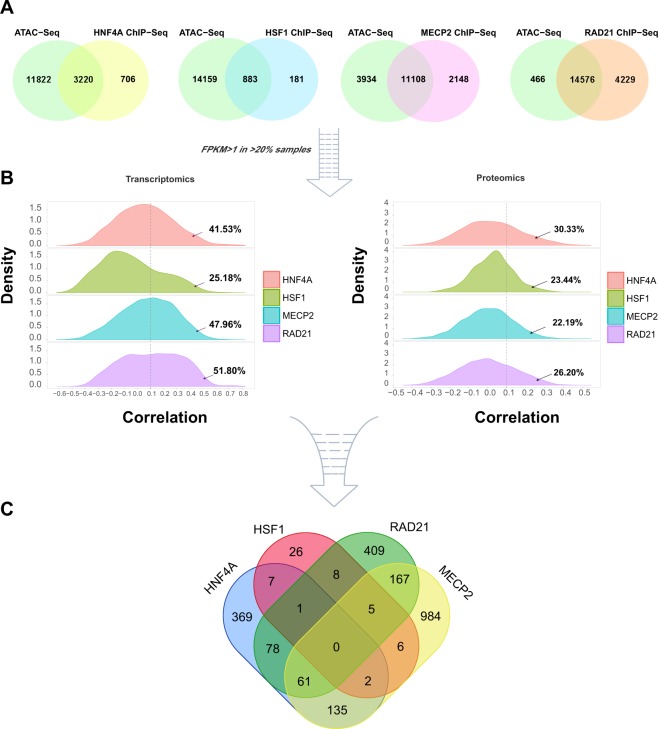
Multi-step screening of the downstream target genes of HNF4A, HSF1, MECP2 and RAD21. (**A**) The Venn diagrams show the intersection of annotated target genes in ATAC-Seq and ChIP-Seq profiles. (**B**) The density plots show the distributions of Spearman correlation coefficients between transcription factors and their target genes at transcriptomics and proteomics levels. The arrows indicate the proportions of target genes with correlation coefficients greater than 0.1. (**C**) The Venn diagram shows the 2258 target genes that ultimately obtained.

After intersecting the annotations between ATAC-Seq and ChIP-Seq profiles, we obtained the lists of putative target genes regulated by HNF4A, HSF1, MECP2 and RAD21 in transcriptional active regions (Fig. 5A).

Besides the above target genes selection based on genomic features, we further used the gene expression information to choose optimization subsets. In detail, we calculated the Spearman correlation coefficients of FPKMs between each TF and their target genes. According to the correlation coefficients distribution of each TF, we set 0.1 as the threshold to screen their optimal target genes (Fig. 5B). Such setting was due to the following reasons: (1) we assumed that there are positive regulatory relationships between transcription factors and target genes, that is, we mainly focused on the transcriptional activation function of these TFs; (2) the threshold (>0.1) allowed top 25% ~ top 50% target genes of each TF to be selected, which is an appropriate range.

Since the relationships between proteins and RNAs are more intuitive, we also calculated the correlation coefficients between the proteomics data of each TF and FPKMs of their target genes in 88 colorectal cancer patients. To be consistent with the above analysis, we chose the same threshold (>0.1), which showed more strict selections for only retaining top 20% ~ top 30% candidates (Fig. 5B). After that, we selected the overlapped target genes based on both RNA and protein expression information as the finally available output of this workflow. At last, 2258 credible target genes of HNF4A, HSF1, MECP2 and RAD21 (Fig. 5C) (Supplementary Table S5) were obtained for downstream analyses.

**Figure 6:**
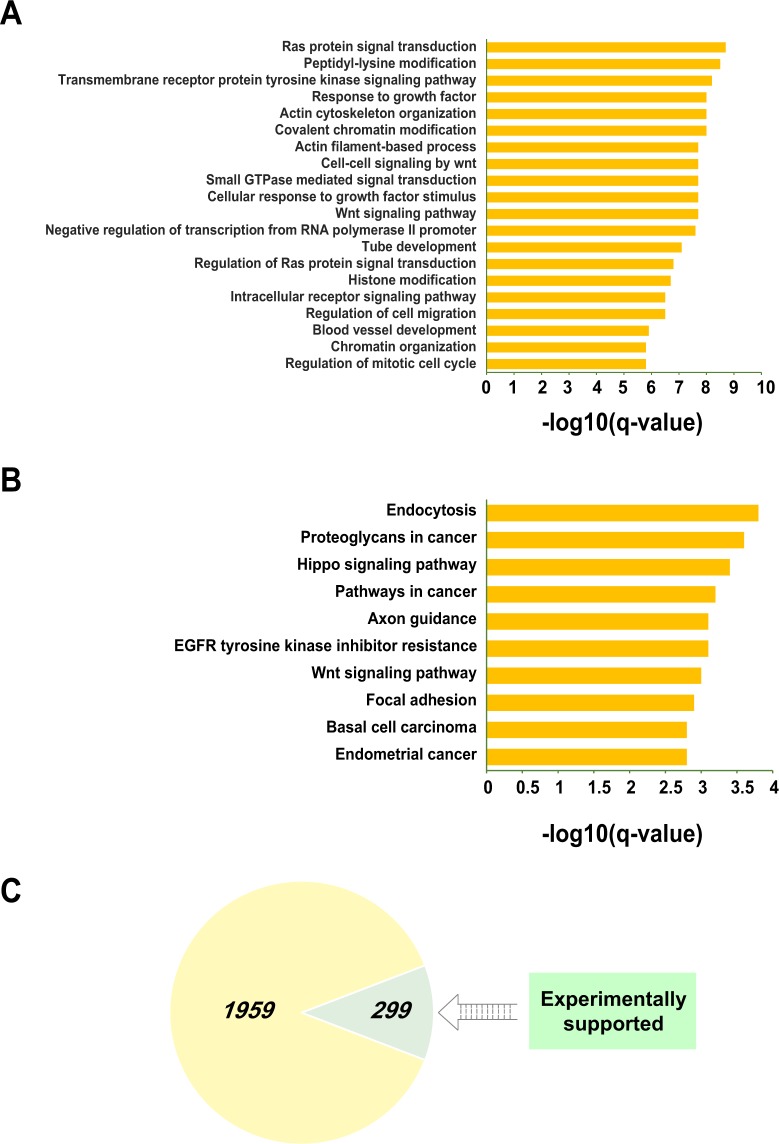
Functional enrichment analyses of 2258 downstream target genes regulated by HNF4A, RAD21, MECP2 and HSF1. The bar plot exhibits top 20 GO annotations and top 10 KEGG signaling pathways according to their −log10(q-values) (**A**,**B**), and the pie chart demonstrates that 299 target genes had been reported to be associated with tumor metastasis from public literatures (**C**).

In order to understand their biological functions, we conducted functional enrichment analyses to these target genes (Supplementary Table S6). As can be seen from Fig. 6A, there was a relatively large part of the top 20 GO annotations related to tumorigenesis and metastasis, such as Ras protein signal transduction, Wnt signaling pathway, response to growth factors, regulation of cell migration, and so forth. Moreover, the top 10 KEGG signaling pathways also showed close relationships with carcinogenesis (Fig. 6B) (Supplementary Table S6). In addition, based on the HCMDB (human cancer metastasis database)^28^, we identified 299 target genes that were proved to play roles in human tumor metastasis from published literatures (Fig. 6C) (Supplementary Table S6).

These evidences revealed the biological functions of the downstream target genes, which also demonstrated the effect of the transcription factors’ dysregulation on colorectal cancer metastasis to some extent.

### Inferring intrinsic associations of target genes based on Bayesian networks

**Figure 7:**
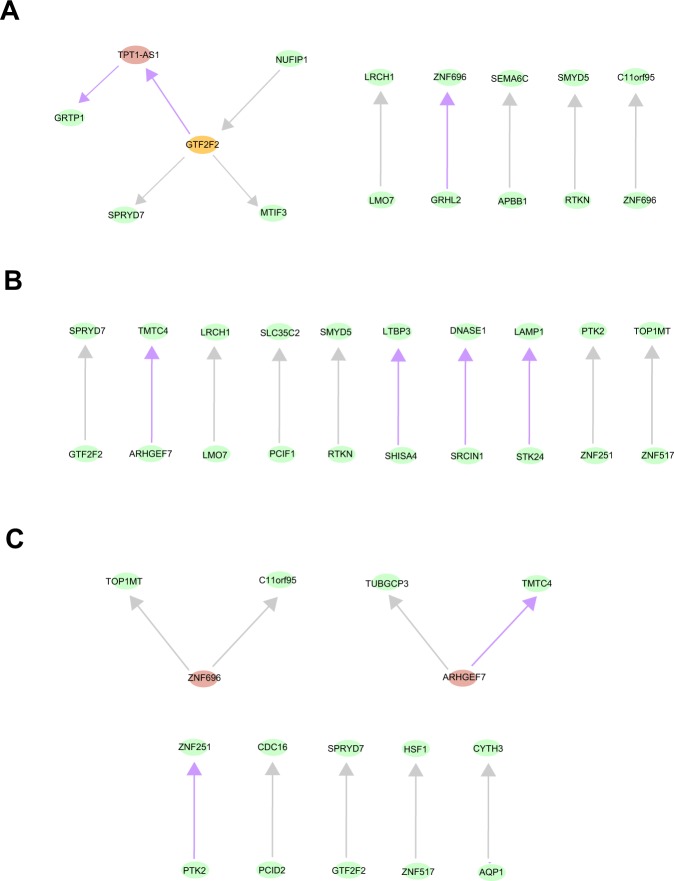
The metastasis–specific Bayesian networks. The network structures were learned from the RNA expression of metastasis-related target genes of HNF4A (**A**), MECP2 (**B**) and RAD21 (**C**) in metastatic patients. The purple arrows represent the relationships specific to metastatic patient group. The grey arrows represent the relationships that belong to both the non-metastatic and metastatic patients group.

The Bayesian network is an advanced method that integrates probability, statistics and graph theory to perform uncertainty reasoning and analysis. In general, a Bayesian network is a directed acyclic graphic model, where each node represents a random variable and each arc represents the probability dependence relationship between two variables. Unlike some previous studies that used the protein-protein interactions to represent the relationships between genes obtained from high-throughput sequencing datasets^29^, the structures of Bayesian networks are directly learned from the gene expression datasets and reflected the probability associations between gene pairs, which are more credible and realistic. However, one obvious limitation of fitting Bayesian networks is the computational complexity that increased exponentially with the number of variables. Therefore, it is necessary to choose the suitable scale of variables and optimal algorithms. Here, we performed multivariate logistic regression (the predictor variables were patients age, gender, and log-transformed FPKM; the response variable was patients metastasis state: metastasis = 1, non-metastasis = 0) on each of the 2258 target genes. In the results, based on the statistical significance (“Benjamini & Hochberg” method adjusted P value < 0.05), we selected 113 gene candidates (40 genes for HNF4A, 47 ones for RAD21, 73 ones for MECP2, 5 ones for HSF1) as the variables in Bayesian networks (Supplementary Table S7). After that, for each TF, through the tabu search-based bootstrap sampling algorithm, we built the Bayesian networks for metastatic and non-metastatic patients, respectively (see Material and Methods). After comparing the two types of network structures, we identified the arcs just belong to the metastatic patient group. From the results, we found that, except HSF1 with no arcs, other transcription factors all had the edges specific to metastatic patients: 30.0% for HNF4A, 40.0% for MECP2 and 22.2% for RAD21 (Fig. 7A–C). On the other hand, for the metastasis-specific Bayesian networks, the structures were sparse and the degrees of nodes were relatively low, that is, the “super nodes” were few. It suggested that there was no obvious centralization and aggregation in the relationships between these important genes, and the nodes evenly participated in the Bayesian networks. Moreover, the directions of the arcs represented the potential casual-effect relationships from a probabilistic perspective.

In summary, we fitted Bayesian networks on the important target genes whose expression values displayed significant effects on colorectal cancer metastasis. Such analyses depicted the underlying collaborations between these genes that may offer a novel view to explore their roles in metastasis.

### Identification of potential metastasis-related biomarkers

In the previous parts, we analyzed the characteristics of these target genes from different angles, including functional enrichment analyses, retrieval of public literatures and metastasis-specific Bayesian networks. Even so, we yet attempted to determine some gene candidates as clinical biomarkers.

**Figure 8:**
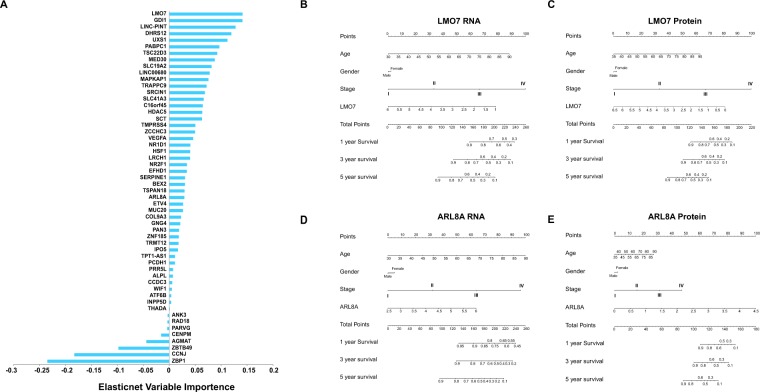
Machine learning approaches and statistical modeling for significant gene candidates selection. 54 gene candidates were obtained through elastic net algorithm and sorted by their non-zero coefficients (**A**). The nomograms based on the multivariate Cox regression models were used to predict the survival probabilities (1-year, 3-year and 5-year) of colorectal cancer patients. Both the RNA and protein expression of LMO7 (**B**,**C**) and ARL8A (**D**,**E**) were incorporated into Cox regression models, which were adjusted by age, gender and tumor stages.

Given that it is one of the advantages of machine learning approaches to extract important features from high dimensional datasets, we implemented elastic net regularized regression on transcriptome profiles of the 2258 target genes. We selected the optimal parameters based on cross validations (see Material and Methods) and gained 54 gene candidates as the significant features for distinguishing the metastatic patients from the non-metastatic group (Fig. 8A) (Supplementary Table S8). Moreover, we also exerted multivariate logistic regression on these gene candidates (gene expression, age and gender were used as predictor variables; non-metastasis = 0 and metastasis = 1 as the response variables). In the results, 51 genes were identified with statistical significance (“Benjamini & Hochberg” method adjusted P value < 0.05), including 45 ones with positive regression coefficients and 6 ones with negative regression coefficients, which implied their adverse impact on metastasis (Supplementary Table S8).

Additionally, considering the closely association between survival status and tumor progression, the effects of these significant genes on the overall survival rate of patients were evaluated through multivariate Cox regression modeling in both RNA and protein levels (the transcriptomic expression values and proteomic expression values were incorporated into Cox regression models as the independent variables individually, and adjusted by gender, age and tumor stages). The results showed that, LMO7 and ARL8A can significantly affect patients’ overall survival rate in both RNA and protein level, whereas, the two genes showed opposite trends (Supplementary Table S8), which suggested their distinct effects, although all intervened in metastasis.

As a kind of useful clinical prediction tool, nomograms were widely applied to evaluate the clinical prognosis of patients. Here, four nomograms were generated from the above multivariate Cox regression models of LMO7 and ARL8A in RNA and protein levels: LMO7-RNA, LMO7-protein, ARL8A-RNA and ARL8A-protein. In these models, the patients survival probabilities can be predicted by summing up the scores of every element, including their clinical information and the expression value of LMO or ARL8A (Fig. 8B–E). Furthermore, to measure the performance of the predictions, we calculated the concordance index (c-index) of each model: 0.725 for LMO7-RNA model, 0.778 for LMO7-protein model, 0.723 for ARL8A-RNA model, 0.811 for ARL8A-protein model (Supplementary Table S8).

All in all, we sought for the appropriate gene candidates rely on different analysis strategies, which may indicate their possible clinical applications.

## Discussion

As other types of cancer, the occurrence of colorectal cancer metastasis is a highly dynamic and multi-step process, including tumor local invasion, moving through the walls of blood vessels, traveling in lymphatic and circulatory system, extravasation and spread to surrounding tissues, tumor angiogenesis and cancer cell proliferation. As long as the physiological conditions are favorable for each step, a new colonization will appear in other tissues of the body. Whereas our knowledge on the intrinsic mechanisms of such clinical phenomenon is still limited.

In our study, we identified four transcription factors associated with colorectal cancer metastasis and implemented integrative analyses to profile their regulatory landscape from genomic levels to clinical phenotypes. After papers review, we found that numbers of previous cancer integrative analyses were mainly based on the high throughput RNA-Seq profiles. However, there is not a simple linear relationship between RNA and protein expression patterns, that is, some other factors can also affect the expression of proteins, such as mRNA stability, mRNA translation efficiency, posttranslational modification and protein degradation. In view of this, we specially integrated both transcriptomics and proteomics datasets of colorectal cancer patients for our analyses, which can form mutual validations and increased the credibility of results.

In our results, copy number variation showed more evident influence on the expression of transcription factors compare to DNA methylation, especially its potential impact on metastasis. However, MECP2 was not sensitive to either of these two genomic variations, which may suggest regulations from other levels.

To be a kind of crucial functional elements, long non-coding RNA (lncRNA) widely participated in regulating the cellular physiological activities in multiple levels.

In particular, lncRNAs can mediate the adjacent protein-coding genes expression in cis-regulatory manner, and the underlying molecular mechanisms are complex and ambiguous^30,31^. Besides that, the direct interaction with proteins is also a subtle regulatory manner of lncRNAs. In our analysis, we screened the lncRNA candidates that may regulate the expression of transcription factors in RNA level and affect their activities in protein level. Based on the multiple bioinformatics analyses, we identified the putative regulatory lncRNA-TF pairs. Such analysis process can be used as an alternative approach to study the lncRNA-mediated regulations or lncRNA-protein interactome for lacking of high-throughput datasets.

Interestingly, the three putative lncRNAs, TPT1-AS1, SNHG15 and LINC01138, which can interact with HNF4A and MECP2 in protein level, had been reported to be related to several human tumors. TPT1-AS1 downregulated in anaplastic gliomas and was associated with the prognosis of patients^32^. LINC01138 was validated as an androgen-responsive lncRNA in prostate cancer^33^. Recently, Li *et*.*al*. found LINC01138 can stabilize PRMT5 through physical interactions in hepatocellular carcinoma and facilitate the proliferation, invasion and metastasis of cancer cells^34^. SNHG15 can interact with EZH2 to repress the expression of P15 and KLF2 in pancreatic cancer, which act as a epigenetic regulator^35^. All of these evidences implied the potential regulatory functions of the three lncRNAs in tumorigenesis and progression. So our results may provide some new hints for exploring their roles in colorectal cancer.

ATAC-Seq, as a powerful technique for detecting chromatin accessibility, has higher sensitivity and accuracy in capturing the chromatin open area compare to MNase-seq and DNase-Seq^36^. For these consideration, the ATAC-Seq and ChIP-Seq datasets of HNF4A, HSF1, MECP2 and RAD21 were integrated to screen high confidence downstream targets in whole genomic level. It should be noted that, we selected the target genes which were activated by these transcription factors, but some transcription factors were multifunction proteins with dual regulatory ability, including both activation and repression. As an example, MECP2 can bind to methylated DNA sequence and repress transcription^37^. However, some studies also demonstrated the transcription activation functions of MECP2 in cells^38,39^. Therefore, although we mainly analyzed the role of each TF in transcription activation, the diversity of their functions should not be ignored.

In the downstream analyses, Bayesian networks were used to discovery the underlying relationships between metastasis-related target genes. In detail, the network structures were learned from target genes expression in both non-metastatic and metastatic patient groups, and the metastasis-specific arcs were also identified for each TF. These results, from a probabilistic point of view, revealed the potential associations between target genes in RNA level, which were more intuitive compare to inferring such relationships from other external evidence (e.g. protein-protein interaction).

Moreover, the elastic net algorithm provided us an optimized subset of gene candidates related to colorectal cancer metastasis. Among them, in both RNA and protein levels, LMO7 and ARL8A showed statistical significance in survival analysis. Despite the role of ARL8A in colorectal cancer was rarely reported, LMO7 had been manifested dysregulation in human cancers. And Nakamura *et al*. found the down-regulation of LMO7 may promote the development of human lung cancer^40^. Besides that, LMO7 significantly upregulated in invasive breast cancer cells, and it can regulate cell migration through mediating the activation of Rho-MRTF-SRF signaling pathway^41^. So our analyses were consistent with the previous studies. Interestingly, according to the results from survival analysis, LMO7 exhibited protective effect on patients survival rate compare to ARL8A. For such phenomenon, we speculated that, despite involved in colorectal tumor metastasis, LMO7 may not be a risk factor from the view of population level.

Admittedly, the limitations that exist in our study should not be neglected. Firstly, although our study integrated multi-omics data from different levels for analyses, the results need to be further verified in independent validation datasets. Secondly, we observed the specific gene expression variations associated with clinical phenotypes of metastatic patients, however, it is not easily to determine the causal-effect relationships between these factors. Given that, the underlying biological significance requires solid evidence from biological experiments and clinical trials.

In summary, our research comprehensively demonstrated the intrinsic functions of four transcription factors in colorectal cancer metastasis, which may extend our knowledge on the underlying mechanisms. In addition, the strategies and methods in our analyses can serve as a paradigm for relevant cancer studies. At last, we trust that our findings can offer new insights into the clinical therapy of colorectal cancer.

## Materials and Methods

### Raw datasets and preprocessing

1. We downloaded the clinical information and RNA-Seq profiles of 598 colorectal cancer (COAD and READ) samples from “National Cancer Institute (NCI) GDC Data Portal”. Among them, 88 samples were derived from patients with tumor metastasis. And we also removed the low expression genes (FPKM > 1 in less than 20% samples).
2. 589 unique masked copy number segment files (Affymetrix SNP 6.0 platform) of COAD and READ patients were obtained from NCI GDC data portal. The GISTIC 2.0 software^15^ was applied to detect significant recurrent copy number variations in all of the tumor samples. In detail, we chose hg38 as reference genome. For the parameter setting: 0.1 as the cutoff of defining the copy number amplification and deletion of genomic regions; 0.99 as confidence level; 0.25 as the significant threshold of q-values. The discretized copy number variation indicators: high deletion = −2; deletion = −1; no change = 0; amplification = 1; high amplification = 2.
3. 371 unique DNA methylation datasets (Illumina Human Methylation 450 platform) of COAD and READ patients were obtained from NCI GDC data portal. Given that each of the four transcriptional factor (HNF4A, HSF1, MEPC2, RAD21) had multiple DNA methylation probes, we chose the ones that most anti-correlated with their corresponding gene expression values (FPKM).
4. ATAC-Seq and ChIP-Seq profiles of HNF4A, HSF1, MECP2 and RAD21 were obtained from Cistrome Data Browser^12^ and the corresponding source data is as follows:
  ATAC-Seq: GSM2579644^42^ HCT-116 cell line
  ChIP-Seq of HNF4A: GSM1242266^43^ LoVo cell line
  ChIP-Seq of HSF1: GSM951880^6^ HT29 cell line
  ChIP-Seq of MECP2: GSM1154509^44^ HCT-116 cell line
  ChIP-Seq of RAD21: GSM1010848^45^ HCT-116 cell line
  Take into account the data quality and the consistency of sources, except HNF4A and HSF1, other datasets were derived from HCT-116 cell line.
5. The quantile-normalized and log-transformed spectral count dataset of 90 colorectal cancer patients obtained from the supplementary information in Bing Zhang *et al*.^11^ A total of 7211 proteins were identified in this datasets and 11 samples were obtained from colorectal cancer metastasis patients according to their clinical information.

### Collection of the transcription factors list on colorectal cancer

Based on the Cistrome Data Browser and Gene Expression Omnibus (GEO), we systematically collected the available ChIP-Seq profiles of 459 transcription factors in human colorectal cells. Their data quality varies greatly due to the different data sources.

### Screening of the putative long non-coding RNAs interacting with transcription factors

We obtained the protein sequences of HNF4A, HSF1, MECP2 and RAD21 from UniProt, and the sequences of long non-coding RNAs were all downloaded from Ensembl genome browser. We used these sequences as input to predict their interactions in *catRAPID omic*^25^, *catRAPID express*^26^ and *RPISeq*^27^. The subcellular localizations of long non-coding RNAs were obtained from lncATLAS^23^, which provided the relative concentration index (RCI) in multiple cell lines for inferring their localizations information.

### ATAC-Seq and ChIP-Seq peaks annotation and visualization

We adopted the R package *ChIPseeker*^46^ for annotating the peaks in ATAC-Seq and ChIP-Seq profiles. In detail, the “TxDb.Hsapiens.UCSC.hg38.knowGene” was used as the annotation database, and the promoter region was defined as ±3 kb around the transcriptional start site (TSS). The annotations of peak profiles were classified into 7 genomic features: promoter, exon, intron, 5′ UTR, 3′ UTR, downstream and distal intergenic. From the results, we removed the peaks annotated in distal intergenic regions, and only retained the ones most related to the nearest genes for further analysis.

### Functional enrichment analyses

For the 2258 downstream target genes, we performed functional enrichment analyses in Metascape^47^. The related gene ontology (GO) and KEGG pathway annotations were ranked based on their −log10(q-value) and we selected the ones with q-value < 0.01.

### Construction of metastasis-specific Bayesian networks for each transcription factor

Through the tabu search-based bootstrap sampling algorithm, for each transcription factor, 500 network structures were learned from the RNA expression of metastasis-related target genes, and the averaged networks whose edges appeared in at least 75% of these networks were selected as the final models. We constructed the Bayesian networks in both non-metastatic and metastatic patient groups and identified the metastasis-specific arcs. The FPKM of each target gene was discretized into three breaks based on its quantile. All of these analyses were performed by *bnlearn* R package.

### Statistical analysis

We performed elastic net algorithm by *glmnet* package in R. In particular, through 10 times 10-fold cross validations, we chose 0.055 as the lambda parameter. In the multivariate logistic and Cox regression modeling, the 2258 target genes expression were transformed into log2 (FPKM+1).

## Electronic supplementary material

Supplementary information

Supplementary Table S1

Supplementary Table S2

Supplementary Table S3

Supplementary Table S4

Supplementary Table S5

Supplementary Table S6

Supplementary Table S7

Supplementary Table S8

## Data Availability

The clinical information, RNA-Seq profiles, copy number variation files, DNA methylation files analyzed during the current study are available in [National Cancer Institute GDC Data Portal] repository, [https://portal.gdc.cancer.gov/] The proteomics datasets analyzed during this study are included in Zhang, B. *et al*. Proteogenomic characterization of human colon and rectal cancer. Nature 513, 382–387, 10.1038/nature13438 (2014). (and its Supplementary Tables). The ATAC-Seq and ChIP-Seq datasets of HNF4A, HSF1, MECP2 and RAD21 analyzed during the current study are available in the [Cistrome Data Browser] repository, [http://cistrome.org/db].
