# Supplementary material for: Genomic and regulatory characteristics of significant transcription factors in colorectal cancer metastasis
Source: Sci Rep. 2018 Dec 13;8:17836. doi: 10.1038/s41598-018-36168-8 (PMC6292939; doi:10.1038/s41598-018-36168-8)
Supplement: Supplementary file 1 — Supplementary information [file 41598_2018_36168_MOESM1_ESM.pdf]

Genomic and regulatory characteristics of significant transcription factors in colorectal cancer metastasis

Bin Zhou[1 *], Rui Guo [2]

[1] School of Life Science, Tsinghua University, Beijing 100084, China

[2] Department of Biochemistry and Molecular biology, Shanxi Medical University, Taiyuan, China

[1] * bin-zhou12@mails.tsinghua.edu.cn

[2] sxykdxgr@139.com

Supplementary Information

Supplementary Figure 1. Genome-wide distributions of ATAC-Seq and ChIP-Seq profiles. (A-D) The heatmap and average profile plots of the ChIP-Seq peaks mapped to ±3kb regions from the transcription start sites (TSS). (E) The heatmap and average profile plot of ATAC-Seq peaks. The pie chart summarizes the genomic annotations of the peaks in the open regions of chromosomes.

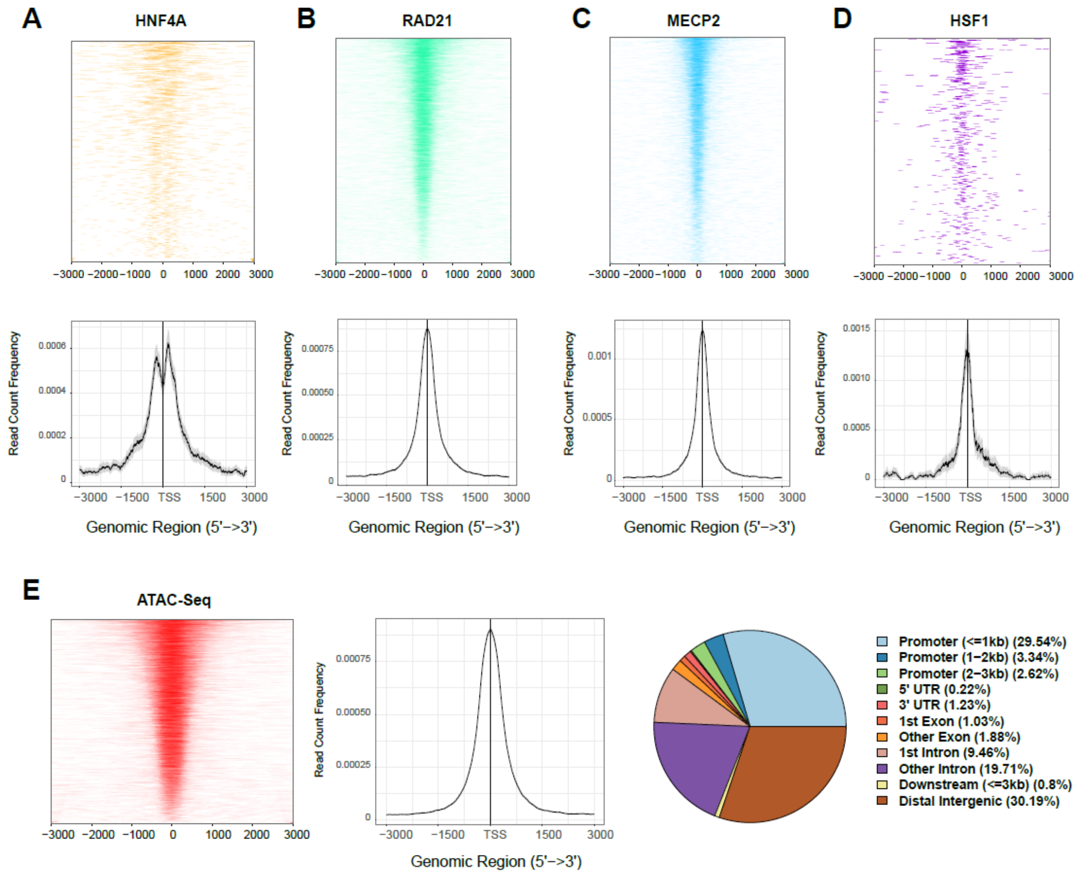

Supplementary Table S1

Wilcoxon rank sum test for identifying differential expressed transcription factors in proteomics and transcriptomics datasets

Supplementary Table S2

Copy number values and DNA methylation beta values of HNAF4A, HSF1, MECP2 and RAD21

Supplementary Table S3

Potential cis-regulatory long non-coding RNAs for transcription factors

Supplementary Table S4

Putative long non-coding RNAs regulating the activities of transcription factors

Supplementary Table S5

Identification of 2258 target genes through multiple step selection

Supplementary Table S6

Functional enrichment analyses and public literature retrieve results

Supplementary Table S7

The Bayesian networks for each TF in non-metastatic and metastatic patient groups

Supplementary Table S8

Elastic net algorithm selection, multivariate logistic and Cox regression results
